# Effects of Isovolumic Loading Elicited Either by 3-Branch or by 4-Branch Spring Expander on the Degree of Cardiac Atrophy of the Failing Heart in Rats After Heterotopic Heart Transplantation: No Evidence for Sex-Linked Differences

**DOI:** 10.33549/physiolres.935650

**Published:** 2025-10-01

**Authors:** Luděk ČERVENKA, Iveta MRÁZOVÁ, Petra ŠKAROUPKOVÁ, Janusz SADOWSKI

**Affiliations:** 1 Center for Experimental Medicine, Institute for Clinical and Experimental Medicine, Prague, Czech Republic

**Keywords:** Heart failure in rats, Cardiac atrophy, Aorto-caval fistula, Heterotopic heart transplantation, Three-branch spring expander, Four-branch spring expander

## Abstract

An important harmful side effect of the prolonged support of the left ventricle (LV) with an LV assist device (LVAD) in patients with advanced heart failure (HF) is development of cardiac atrophy. Our first aim was to evaluate if implantation of the four-branch spring expander into LV of the rat will exhibit greater attenuation of unloading-induced cardiac atrophy as compared with the three-branch spring expander. The second aim was to assess if sex-related differences are present in the development of unloading-induced cardiac atrophy in the failing hearts with implanted either three-branch or four-branch spring expander into the LV. Heterotopic heart transplantation in the rat (HT_x_) served as the model of heart unloading after LVAD implantation. HF was induced by volume overload achieved by creation of the aorto-caval fistula. The degree of cardiac atrophy was assessed as the weight ratio of the heterotopically transplanted heart to the control native heart. We found that enhancement of isovolumic loading by implantation of either type of spring expander into the LV reduced the degree of post-HT_x_ cardiac atrophy in the failing hearts but the four-branch variant was significantly more effective. In addition, we found that there were no sex-related differences in the development of unloading-induced cardiac atrophy or in the attenuation of this process in the failing hearts. We propose that enhancing cardiac work by increasing isovolumic loading *via* implantation of the spring expander might be a reasonable approach to attenuate the unloading-induced cardiac atrophy in the failing hearts in both sexes.

## Introduction

Heart failure (HF) is a global health problem and the progress of this clinical syndrome results in significant morbidity and mortality [1,2]. According to the most recent annual report of the American Heart Association (prepared in conjunction with the National Institute of Health) 6.7 millions of Americans ≥20 years of age had HF in 2024 and the number is expected to increase further, to reach >8 millions by 2030, which will represent 3.0 % of the total population of the United States of America [3]; a similar situation is reported for the European Union [4]. In patients with HF, the left ventricle (LV) undergoes complex adverse molecular, cellular and structural changes that have been described as “LV remodeling”, leading ultimately to further impairment of LV function and progression of HF. It has been claimed that the process is irreversible and inevitably leads to the end-stage HF and consequently to the fatal end [4–9]. Studies of patients with end-stage HF with implanted LV assist device (LVAD) have shown that LVAD-induced unloading of LV was associated with reversal of the pathological cellular, molecular and electro-physiological abnormalities in the myocardium: this process was named “LV reverse remodeling” [10–13]. It has been claimed this “LV reverse remodeling” leads to the LV functional recovery eventually enabling the weaning from LVAD treatment. Those patients could subsequently receive “only” standard pharmacotherapy [14–19]. However, for unknown reasons the biological signs of “LV reverse remodeling” only very rarely were translated into LV functional improvement that would result in LVAD explantation [16,20–28]. One major reason for the divergence between biological characteristics and clinical outcomes was perhaps that cardiac atrophy (consequence of long-term implantation of LVAD) offsets the beneficial effects on biological traits and precludes improvement of LV function [22–24,27–32]. This notion has been evaluated in the experimental studies employing the appropriate model of heterotopic heart transplantation (HT_x_) in the rat. Attempts were made to prevent or at least to minimalize unloading-induced cardiac atrophy after heterotopic HT_x_, but almost all of them, including ours, were not successful [22,27,28,31–36]. Some exception was our study showing enhancement of cardiac work in heterotopically transplanted heart by implantation of the three-branch spring expander. Nevertheless, this approach was not able to fully prevent unloading-induced cardiac atrophy in the failing hearts (i.e. hearts taken from animals with developed advanced HF) after heterotopic HT_x_ [37,38], in contrast to the observations from the pioneer study by Klein *et al.* [39].

More than three decades ago Klein and co-workers [39] placed in the LV of transplanted rat heart an inflated latex balloon that was able to radically increase cardiac work and prevent the development of cardiac atrophy after HT_x_. Unfortunately, this approach cannot be applied in the clinic because in patients with LVAD this procedure would cause obstruction of LV and failure of LVAD function. We assume that the difference in the degree of attenuation of unloading-induced cardiac atrophy elicited by latex balloon vs. three-branch spring expander is caused by the different level of increased isovolumic loading that is produced by these two different procedures. To test this notion, we developed a four-branch spring expander that, according to *in vitro* evaluation of elastic and plastic mechanical deformation properties, should elicit greater isovolumic loading as compared with the three-branch spring expander; application of the four-branch variant still does not affect the LV ejection function.

Considering the above evidence, our ***first aim*** was to examine if the four-branch spring expander will exhibit higher degree of attenuation of unloading-induced cardiac atrophy after HT_x_ compared with the three-branch variant. It is important to recognize that the limitation of all our previous studies evaluating the process of unloading-induced cardiac atrophy in the failing hearts was observed with our standard model of heterotopic HT_x_. This means that the failing hearts were before HT_x_ chronically exposed to marked activation of various neurohormonal systems that characteristically accompany the advanced phase of HF [40,41] and then were suddenly placed in normal neurohormonal environment of healthy animals (for technical reasons healthy recipients are standardly employed in the model of heterotopic HT_x_ [27,28,30–35,37–39,42]). Such abrupt change in hormonal environment could have some modulatory action on the degree of unloading-induced cardiac atrophy and particularly on the effects of expander implantation on this process. Therefore, to overcome this limitation in the current study we have decided to use as the recipients the animals at the advanced stage of HF (at the same phase of HF as that of the failing donor hearts used).

Since there are still uncertainties about the potential role of sex-linked differences in the process of unloading-induced cardiac atrophy in the failing hearts [38] and to follow recent recommendations indicating that “sex” should no longer be an ignored experimental variable, the ***second aim*** of the present study was to evaluate if sex-related differences modify in the degree of unloading-induced cardiac atrophy in the failing hears with implanted three-branch or four-branch spring expander into LV. There is no doubt that sex is an important parameter in preclinical research, and should be considered for successful translation of results into clinical practice [43,44].

## Methods

### Ethical approval

The studies were performed in agreement with the guidelines and practices established by the *Animal Care and Use Committee of the Institute for Clinical and Experimental Medicine*, Prague, which accord with the *European Convention on Animal Protection and Guidelines on Research Animal Use* and were approved by this committee and subsequently by the Ministry of Health of the Czech Republic (the decision number for this project is MZDR 6166/2025-5/OVZ).

### Animals, HT_x_ and HF models

Adult male and female Lewis rats (Charles River Laboratories, Velaz, Prague, Czech Republic), 8 weeks of initial age, were used. The classical heterotopic HT_x_, originally described by Ono and Lindsey [42] and employed and validated by many investigators was used as the model to simulate the effect of significant mechanical unloading of the heart; its modification was established in our laboratory and is routinely employed [27,28,30–39]. HF was induced by volume overload induced by aorto-caval fistula (ACF) created using needle technique as originally described by Garcia and Diebold [45] and then employed and validated by many investigators including our own group [46–52]. Eight weeks after ACF creation the animals were used as heart donors and their littermates prepared in the same way (i.e. with ACF) were used as recipients for heterotopically transplanted failing hearts. Earlier studies, including ours, demonstrated that at that time ACF animals are in the stage of advanced HF and if untreated soon progress toward decompensated hypertrophy and HF [46–52].

### Spring expanders

**Fig. 1:**
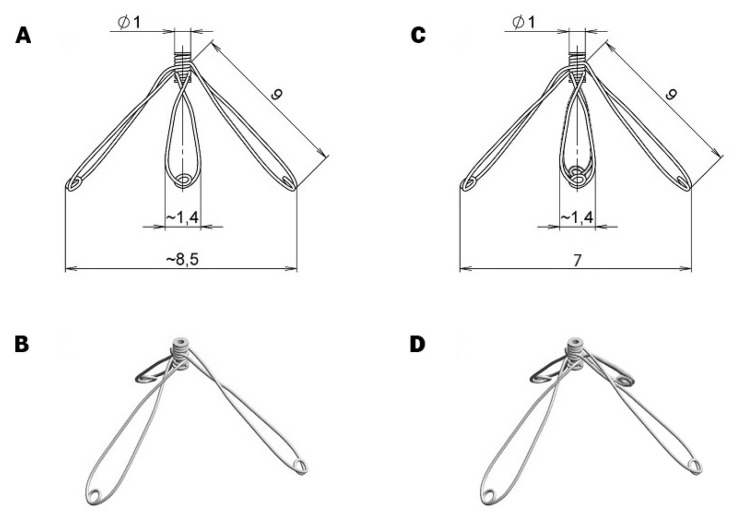
Diagrammatic presentation of the three-branch expander (**A**) and four-branch expander (**C**) and the general view of the three-branch expander (**B**) and four-branch expander (**D**).

The stainless steel three-branch spring expander with the branch length of 9 mm at the same composition of the stainless wire as employed in our previous studies was used in the present study (Fig. 1A, B). In addition, the novel four-branch spring expander with the same branch length of 9 mm and same composition of the stainless wire was used in the present study (Figs 1C, D). Elastic and mechanical properties of both spring expanders were measured *in vitro* on the miniaturized compression device and analyzed by generation of stress-strain relationship as described by Lossef *et al.* [53]. According to this *in vitro* analyses and subsequent computer modeling isovolumic loading in accordance with recent progress in this field [54–56] we obtained the data suggesting that four-branch spring expander should increase the isovolumic loading and consequently cardiac work by approximately 17 % as compared with three-branch variant.

### Experimental design

#### Comparison of enhanced isovolumic loading induced either by three-branch or four-branch spring expander implantation into the LV on the cardiac atrophy after heterotopic HT_x_ in failing hearts

HT_x_ of the failing heart was performed and, in appropriate groups, implantation into the LV of either stainless steel three-branch expander (briefly: “three-branch expander”) or stainless steel four-branch expander (briefly: “four-branch expander”) was performed through LV apex incision. We and others [23,27,28,31,33,34,37] have demonstrated that the unloading-induced cardiac atrophy develops within the first 14 days after HT_x_ when a dramatic loss of myocardial mass is seen. The following 40 days is a steady-state period, with no further loss of cardiac mass, suggesting stabilization of unloading-induced cardiac atrophy. Therefore, in the present study the degree of cardiac atrophy was assessed 14 days after HT_x_. The degree of atrophy was assessed from the weight of the total heart and its individual structural components [LV + septum and right ventricle (RV)]. Explicitly, the index of cardiac atrophy was calculated as the ratio of the weight of the heterotopically transplanted heart to the recipient native failing heart. The degree of cardiac atrophy was expressed as percent decrease in the whole heart weight (HW), LV weight (LVW), and RV weight (RVW) of the hearts after HT_x_. Unfortunately, HW of the donor’s heart before and after HT_x_ cannot be used for evaluation of the degree of cardiac atrophy, because the heart is immediately placed in cold cardioplegia solution, which precludes precise determination of HW. Therefore, hearts from littermates prepared in the same way as described above served as basal values (100 %) for evaluation of the process of cardiac atrophy after HT_x_. The following groups were examined (n=11 in each):

1. ACF (10 weeks after creation of ACF) male Lewis rats (recipient) + HT_x_ of failing male donor’s heart (14 days after HT_x_),
2. ACF male Lewis rats + HT_x_ of failing male donor’s heart + implantation of three-branch expander,
3. ACF male Lewis rats + HT_x_ of failing male donor’s heart + implantation of four-branch expander,
4. ACF female Lewis rats + HT_x_ of failing female donor’s heart,
5. ACF female Lewis rats + HT_x_ of failing female donor’s heart + implantation of three-branch expander,
6. ACF female Lewis rats + HT_x_ of failing female donor’s heart + implantation of four-branch expander,

At the end of the experiment the hearts were excised, blood was removed from the chambers by gentle compression, and the hearts’ wet weight was determined.

### Statistical analyses

All values are expressed as mean ± SEM. Using the Graph-Pad Prism software (Graph Pad Software, San Diego, CA, USA), statistical analysis was done by Wilcoxon’s signed-rank test for unpaired data, or one-way analysis of variance (ANOVA) when appropriate. The values exceeding 95 % probability limits (p<0.05) were considered statistically significant.

## Results

Table 1 collects the absolute values of whole HW, LVW and RVW of the native failing hearts (served as basal value representing 100 % for evaluation of the degree of cardiac atrophy) and of transplanted failing hearts obtained 14 days after HT_x_.

Table 2 summarizes the body weights (BW), tibia lengths (TL) and HW and LVW either normalized to BW or TL of the native failing hearts. As shown, when normalized to TL, male organs exhibited higher cardiac mass and, in contrast, if normalized to BW, female organs showed higher cardiac mass.

**Fig. 2:**
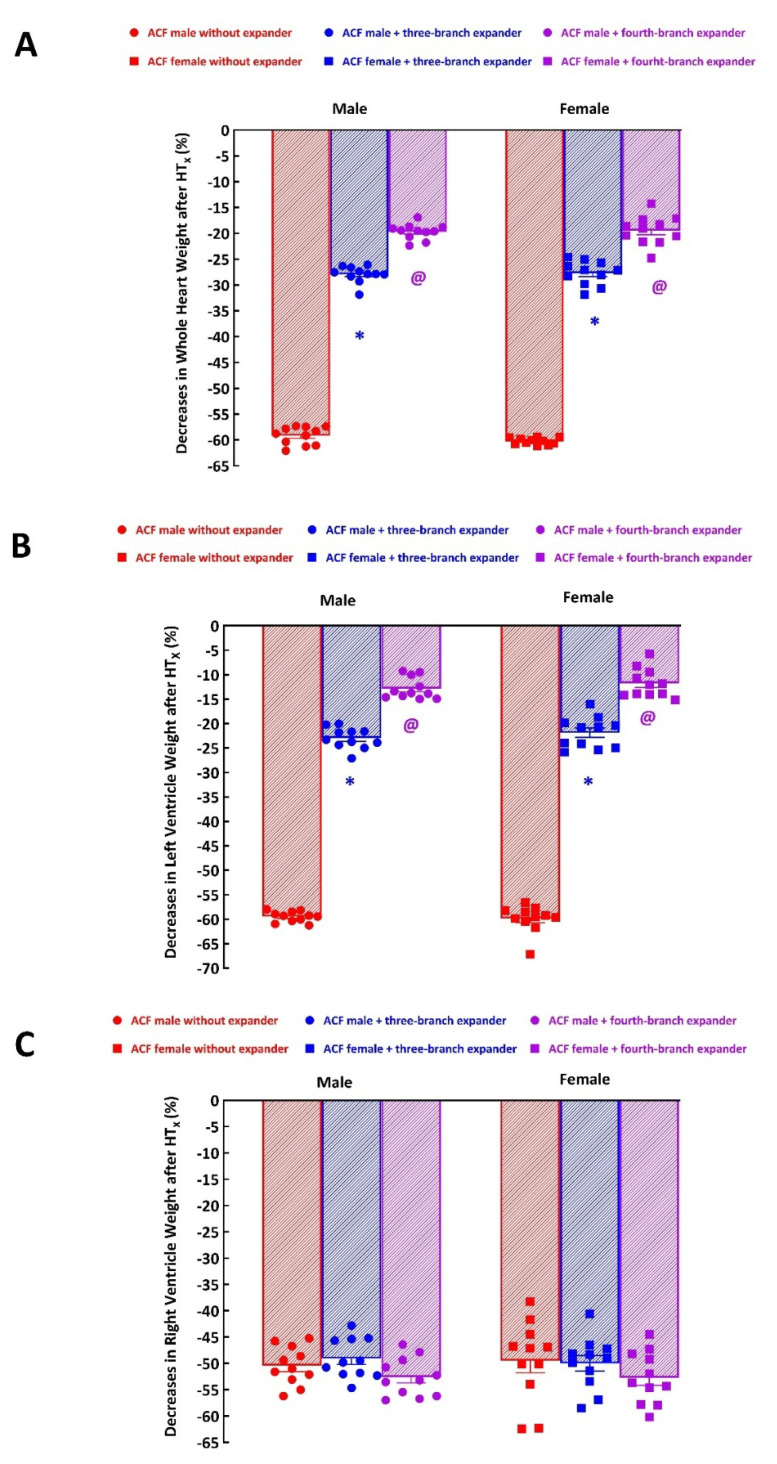
Effects of implantation of either the three-branch or the four-branch spring expander on the course of cardiac atrophy in response to mechanical heart unloading induced by heterotopic heart transplantation (HT_x_) in male and female Lewis rats with heart failure elicited by creation of the aorto-caval fistula (ACF). Data are expressed as percent decreases compared with the native failing heart: (**A**) changes in whole heart weight, (**B**) changes in left ventricle weight, (**C**) changes in right ventricle weight. *p<0.05 compared with animals without the expander. ^@^p<0.05 compared with animals with implanted three-branch spring expander.

As shown in Figure 2A, 14 days’ unloading by HT_x_ elicited in failing hearts profound but similar decreases in whole HW in male and female ACF rats (−59±1 and −60±1 %, p>0.05). Implantation of three-branch expander significantly reduced the decreases in whole HW in ACF male rats (−28±1 vs. 59±1 %, p<0.05) as well as in ACF female rats (−28±1 vs. −60±1 %, p<0.05). Implantation of four-branch expander further attenuated the decreases in HW after HT_x_ and the decreases were significantly less pronounced than in those with three-branch expander implantation in ACF male rats (−20±1 vs. −28±1 %, p<0.05) as well as in ACF female rats (−19±1 vs. −28±1 %, p<0.05). The degree of LVW and RVW decreases in ACF male rats and in ACF female rats without implantation of spring expander were quite similar as those observed in whole HW (Fig. 2B, C). Implantation of either three-branch or four-branch spring expander caused similar attenuation of decreases in LVW as that observed in whole HW (Fig. 2B). The implantation of expanders did not have any significant effect on HT_x_-induced RVW decreases in ACF male rats as well as in ACF female rats (Fig. 2C).

## Discussion

***The first important set of findings*** of the present study relates to our observation that the enhancement of isovolumic loading induced by implantation of both types of spring expander into the LV significantly attenuated the degree of unloading-induced cardiac atrophy in the failing hearts. The degree of cardiac atrophy as well as its reduction was essentially the same in male and female rats.

However, of critical importance here is the observation that implantation of four-branch spring expander was significantly more effective in attenuating the unloading-induced cardiac atrophy after HT_x_ in the failing hearts as compared with the implantation of three-branch expander. This finding confirms our assumption that the extent of attenuation of unloading-induced cardiac atrophy after HT_x_ depends on the level of enhanced cardiac work elicited by increased isovolumic loading obtained by implantation of the expander. It also supports our explanation why placing of inflated latex balloon in the LV of transplanted heart was able to fully prevent the development of unloading-induced cardiac atrophy, as reported from an early study [39].

The second important observation is related to the divergence of our results in the failing hearts when compared with previous results obtained in the healthy (non-failing) hearts. Our previous studies in the healthy hearts demonstrated that: first, that cardiac atrophy in the healthy hearts is distinctly less pronounced than in the failing hearts [33,34,37]. Second, implantation of the spring expander (three-branch) did not attenuate the development of post-HT_x_ cardiac atrophy in the healthy hearts [34]. One explanation of the divergence between the healthy hearts and the failing hearts might be different neurohormonal environment and its potential modulatory actions on the course of unloading-induced cardiac atrophy after HT_x_ as was proposed recently [38]. It is generally accepted that only the healthy rats can be used as recipients for HT_x_, because the animals with advanced HF (8 to 10 weeks after ACF creation) would not survive demanding surgical procedure of HT_x_ (perioperative and postoperative mortality would be unacceptably high). Therefore, the hearts from different ACF animals that were prepared in the same way as donors at an appropriate time point served as control basal values (100 %) for evaluation of the process of cardiac atrophy after HT_x_ with the failing hearts transplanted and the recipients being healthy animals [33,34,38]. Apparently, the issue of abrupt change in hormonal environment and its potential effects on the course of unloading-induced cardiac atrophy after HT_x_ was disregarded. An improvement of the surgical technique and postoperative care achieved by us over the last decade enabled us to use recipient animals with the stage of HF the same as that of donor’s hearts taken. In fact, the perioperative and acute postoperative mortality was in the current study 37 %, which is essentially the same as observed when the healthy animals were employed as recipients, because the average perioperative and acute postoperative mortality in our previous studies was 41 % [33,37,38]. Our present findings show that the course of development of cardiac atrophy after HT_x_ in the failing hearts without implantation of the expander and with implanted three-branch expander to the recipients with established advanced HF was essentially the same as observed when healthy animals served as recipients [37,38]. In fact, our present findings exhibited lesser variability. This probably was the result of more consistent control basal values that are obtained from the native failing hearts under conditions of this experimental setup than those obtained from separately prepared ACF animals; this was so despite the fact that the rats were appropriately time-matched [37,38]. Therefore, our present data strongly suggest that differences in neurohormonal environment are not a critical factor in the process of unloading-induced cardiac atrophy after HT_x_ and cannot be responsible for the augmented cardiac atrophy in the failing hearts as compared with the healthy hearts [33,37,38,57,58]. Consequently, the reason might rather be that proposed in our recent study [38]: in the case of the failing hearts more pronounced unloading-induced cardiac atrophy (vs. that observed in the healthy hearts) is the consequence of higher initial baseline levels for heart weights in ACF animals as compared with healthy animals (i.e. without ACF). In other words, as originally proposed by Wilder for similar circumstances and identified as “the law of initial values” [59], augmented decreases in HW, LVW and RVW might be the consequence of the fact that values of those parameters for ACF animals were more than twice higher than those observed in the healthy animals [33,34,37,38,57,58]. Therefore HT_x_-induced cardiac unloading elicited higher percent decreases for failing hearts than those for the healthy hearts.

***The second important set of findings*** of the present study relates to our observation that there were no important sex-linked differences in the degree of unloading-induced cardiac atrophy in the failing hearts and their responses to implantation of spring expanders. This is in agreement with our recent results [38] and the information is now importantly expanded: we show that the absence sex-related differences in the process of cardiac atrophy after HT_x_ in the failing hearts is confirmed under all conditions of isovolumic loading (i.e. without expander implantation and after implantation of both types of expanders). As discussed above, this situation is not modified by alterations of neurohormonal environment as hypothesized in our previous study [38]. Admittedly, this means that we cannot provide any reasonable explanation for the discrepancies observed during evaluation of the extent of post-HT_x_ cardiac atrophy in the healthy hearts. In fact, we repeatedly demonstrated important sex-related differences in the process of unloading-induced cardiac atrophy in the healthy hearts and found that these differences are due to inherent properties of the donor’s heart and cannot be simply ascribed to direct actions of sex hormones [57,58]. Nevertheless, before making such ultimate conclusion, the classical experimental approach (i.e. comparison of intact animals with those after gonadectomy) should be further confirmed by studies investigating the response of the failing female heart transplanted into a male recipient with advanced stage of HF (i.e. at the same phase of HF as that of failing donor heart used). Noteworthily, we demonstrated in our recent study [58] that this method is probably the optimal approach to evaluate the direct actions steroid hormones on the degree of unloading-induced cardiac atrophy. Further studies are needed to complete the classical experimental approach by this cross-sex transplantation approach, which, however, is technically very demanding. Therefore, we can currently only say that the results of our recent [38,57,58] and the present studies clearly show that there are important sex-linked differences in the process of unloading-induced cardiac atrophy in the healthy hearts but none are seen in the failing hearts.

Considering all the pertinent earlier information [33,34,37,38,57,58] and the present results our firm opinion is that, to be useful in patients with implanted LVAD, experimental studies of unloading-induced cardiac atrophy and of potential anti-atrophic measures, should be performed in the failing hearts. In addition, the recipients in the model of heterotopic HT_x_ should preferably be the animals with advanced HF.

## Conclusion, Merits and Perspectives

The results of the present study show that, first, enhancement of isovolumic loading induced by implantation of both types of spring expanders into the LV attenuated the process of post-HT_x_ cardiac atrophy in the failing hearts. However, the four-branch spring expander was significantly more effective than the three-branch variant. Second, we found that there were no sex-related differences in the development of unloading-induced cardiac atrophy or in the attenuation of this process in the failing hearts. We propose that enhancing cardiac work by increasing isovolumic loading *via* implantation of the spring expander might be a reasonable approach to attenuate the unloading-induced cardiac atrophy in the failing hearts of both sexes.

## Figures and Tables

**Table 1:** The weight of transplanted (i.e. donor) heart and the native heart and of the individual heart structural components after heterotopic heart transplantation (HT_X_). Native heart values served as basal values (100 %) for evaluation of the process of cardiac atrophy in animals after HT_x_.

	Parameter					
	HW (mg)(native)	HW (mg)(HT_x_)	LVW (mg)(native)	LVW (mg)(HT_x_)	RVW (mg)(native)	RVW (mg)(HT_x_)
*Group of males*						

*ACF male recipient + HT* *_x_* * of failing male donor’s heart without expander*	2097 ± 5	856 ± 12*	1218 ± 4	494 ± 5*	466 ± 3	232 ± 6*
*ACF male recipient + HT* *_x_* * of failing male donor’s heart + implantation of 3-branch expander*	2085 ± 12	1502 ± 10#	1229 ± 5	947 ± 6#	475 ± 3	225 ± 6*
*ACF male recipient + HT* *_x_* * of failing male donor’s heart + implantation of 4-branch expander*	2093 ± 15	1681 ± 12@	1221 ± 5	1064 ± 7@	468 ± 3	238 ± 5

*Group of females*						

*ACF female recipient + HT* *_x_* * of failing female donor’s heart without expander*	1573 ± 8*	626 ± 5*	963 ± 7	386 ± 7*	349 ± 3	177 ± 9*
*ACF female recipient + HT* *_x_* * of failing female donor’s heart + implantation of 3-branch expander*	1592 ± 8	1151 ± 11#	965 ± 6	754 ± 10#	361 ± 4	171 ± 5*
*ACF female recipient + HT* *_x_* * of failing female donor’s heart + implantation of 4-branch expander*	1586 ± 9	1278 ± 11@	960 ± 5	847 ± 8@	356 ± 5	178 ± 7*

Values are means ± SEM. ACF, aorto-caval fistula; HT_x_, heterotopic heart transplantation; HW, heart weight; LVW, left ventricle weight; RVW, right ventricle weight.

* p<0.05 compared with values from native hearts at the same experimental groups of male and female rats.

# p<0.05 compared with values from native hearts at the same experimental groups of male and female rats and with values hearts after HT_x_ without expander.

@ p<0.05 compared with values from native hearts at the same experimental groups of male and female rats and with values hearts after HT_x_ with implanted 3-branch expander.

**Table 2:** Body weights, tibia length of the native failing heart and normalization of whole heart weight and left ventricle weight (including septum) to body weight and tibia length.

	Parameter					
	BW(g)	TL(mm)	HW/BW(mg/g)	HW/TL(mg/mm)	LVW/BW(mg/g)	LVW/TL(mg/mm)
*Group of males*						

*ACF male recipient + HT* *_x_* * of failing male donor’s heart without expander*	401 ± 3*	39.9 ± 0.07*	5.24 ± 0.04	52.52 ± 0.16*	3.04 ± 0.02	30.50 ± 0.11*
*ACF male recipient + HT* *_x_* * of failing male donor’s heart + implantation of 3-branch expander*	397 ± 2*	40.0 ± 0.13*	5.25 ± 0.04	52.15 ± 0.41*	3.09 ± 0.02	30.85±0.11*
*ACF male recipient + HT* *_x_* * of failing male donor’s heart + implantation of 4-branch expander*	400 ± 3*	40.1 ± 0.16*	5.23 ± 0.05	52.25 ± 0.41*	3.05 ± 0.02	30.48 ± 0.16*

*Group of females*						

*ACF female recipient + HT* *_x_* * of failing female donor’s heart without expander*	272 ± 6	36.1 ± 0.05	5.83 ± 0.04#	43.69 ± 0.35	3.57 ± 0.07#	26.75 ± 0.23
*ACF female recipient + HT* *_x_* * of failing female donor’s heart + implantation of 3-branch expander*	276 ± 2	36.1 ± 0.28	5.77 ± 0.03#	44.16 ± 0.23	3.47 ± 0.02#	26.68 ± 0.10
*ACF female recipient + HT* *_x_* * of failing female donor’s heart + implantation of 4-branch expander*	277 ± 2	36.2 ± 0.10	5.74 ± 0.04#	43.83 ± 0.28	3.48 ± 0.02#	26.52 ± 0.11

Values are means ± SEM. ACF, aorto-caval fistula; HW, heart weight; LVW, left ventricle weight.

* p<0.05 compared with values from female groups, always compared with the same experimental group of female rats.

# p<0.05 compared with values from male groups, always compared with the same experimental group of male rats.
